# Psychosocial Health Status of Persons Seeking Treatment for Exposure to Libby Amphibole Asbestos

**DOI:** 10.5402/2011/735936

**Published:** 2011-05-26

**Authors:** Clarann Weinert, Wade G. Hill, Charlene A. Winters, Sandra W. Kuntz, Kimberly Rowse, Tanis Hernandez, Brad Black, Shirley Cudney

**Affiliations:** ^1^College of Nursing, Montana State University, P.O. Box 173560, Bozeman, MT 59717-3560, USA; ^2^College of Nursing, Montana State University, 32 Campus Drive no. 7416, Missoula, MT 59812, USA; ^3^Robert Wood Johnson Foundation Nurse Faculty Scholar and College of Nursing, Montana State University, 32 Campus Drive no. 7416, Missoula, MT 59812, USA; ^4^Center for Asbestos Related Disease, 214 E 3rd Street, Libby, MT 59923, USA

## Abstract

A cross-sectional exploratory study was conducted to describe the psychosocial health status of persons seeking health care for exposure to Libby amphibole asbestos (LAA). Health indicators including depression, stress, acceptance of illness, and satisfaction with access and financial aspects of care were obtained via electronic and paper-pencil survey. The exposure pathway and demographic data were gleaned from the health record. Of the 386 participants, more than one-third (34.5%) demonstrated significant levels of psychological distress. The oldest group of women had the lowest levels of depression and stress and the highest acceptance of illness. Gender, age, and satisfaction with financial resources were significantly related to depression, stress, and acceptance of illness. Satisfaction with access to care was significant only for stress. No differences in depression, stress, and acceptance of illness were found based on residence, exposure pathway, or insurance status.

## 1. Introduction

Professional response to the psychological needs of individuals exposed to acute, sudden, and catastrophic natural disasters or terrorism has been the focus of recent research [1] and has become an essential component of comprehensive disaster relief services [2]. However, the human emotional response, resiliency, and potential for chronic psychopathology appear to be different when the disaster is a slow motion, technologically associated event with unremitting long-term impacts on the ecosystem, socioeconomic structure, and health of the resident population [3]. For example, the recent Gulf Coast oil-spill disaster in the United States (USA) provides a glimpse into the lives of individuals and communities dealing with the early stages of psychosocial stress associated with a massive, long-term environmental event. Another case in point, and the focus of this paper, is the delayed psychosocial consequences of a complex and persistent environmental event that is being faced by the community and residents of Libby, Montana in the USA. 

Libby, a small rural mountain town (population 2600), was contaminated with amphibole asbestos as a result of decades of mining tainted vermiculite ore. In 2002, with the resolve of politicians, medical providers and community members, Libby was placed on the National Priorities List as a superfund site by the United States Environmental Protection Agency (EPA). The community-wide environmental and occupational contamination left Libby with a legacy of economic and insidious health-related problems across generations and a reputation as a place with “air that kills” [4]. In 2009, seven years after designation as a superfund site, the EPA declared Libby a public health emergency, the first in USA history resulting from an environmental disaster under the Comprehensive Environmental Response, Compensation, and Liability Act (CERCLA). This determination recognized the serious impact to the public health from the contamination at Libby and laid the foundation for a short-term grant to provide needed asbestos-related medical care to persons who were exposed to Libby amphibole asbestos.

Exposure to microscopic asbestos fibers is linked to several malignant and nonmalignant illnesses including lung cancer, mesothelioma, asbestosis, pulmonary fibrosis, and pulmonary effusions [5]. Persons at risk for asbestos-related diseases (ARD) include (1) former vermiculite workers, (2) household contacts and family members who lived with the workers, (3) community members with no association to the vermiculite mine or mine facilities that lived, worked, or played in Libby, and (4) persons living in other communities across the USA where the ore was processed [6]. During 2000-2001, medical screenings were conducted by the Agency for Toxic Substances and Disease Registry (ATSDR) of 7,307 persons who had lived, worked, or played in Libby for 6 months prior to closing of the vermiculite mine (December 31, 1990). Results showed that 18% of persons had pleural abnormalities [7, 8]. Significant increases in asbestos-related pleural abnormalities were also found among residents living in Libby after 1990 who were never associated with the mining operation. Standardized mortality ratios (SMRs) for asbestosis in persons who lived in the city of Libby and surrounding areas were 40–80 times higher than those for Montana and the USA, respectively [9]. 

In the latest asbestosis mortality statistics, Lincoln County, where Libby is located, had the highest age-adjusted asbestosis death rate per million people in the USA for residents age 15 and older [10]. As of March 2010, nearly 325 Libby residents have died from ARD; thirty-two deaths have been attributed to malignant mesothelioma, a rare form of cancer most commonly associated with exposure to asbestos [11]. Because ARD has a latency of 10 or more years, more cases are expected to surface [12]. However, the impact of Libby amphibole asbestos (LAA) extends well beyond this rural community. Tainted vermiculite ore was distributed to over 250 processing and distribution plants across the USA, contributing 80% of the world's supply [13]. It is estimated that the raw ore contained as much as 26% asbestos [7, 14]. Vermiculite is widely used in agriculture, industry, horticulture, and construction [15]. It is estimated that 35 million homes across the USA alone are insulated with the contaminated vermiculite (Zonolite) that came from Libby. The first screening of USA workers exposed to Libby vermiculite products and living outside Libby was carried out in Maryville, Ohio [16] with a follow-up study conducted 20 years later [17]. Pulmonary findings from the Maryville study were similar to the Libby findings from the 2000-2001 health screening.

In addition to physical and ecologic damage, technological disasters can affect the psychosocial health of individuals, families, and communities. Anxiety, chronic stress, depression, loss of control, and uncertainty among disaster victims have been documented [18], while communities can be left with an aftermath of controversy, conflict, and social divisiveness [19]. It is reasonable to expect that living with the knowledge of life-threatening risk to health and the potential for continued exposure, as the EPA cleanup activities continue, would increase Libby residents' vulnerability to depression and stress. While no one is immune, younger age groups and women have been found to be particularly vulnerable to the psychosocial fallout of an environmental disaster [20]. 

Depression results in sadness, loss of interest in a person's usual activities, feelings of worthlessness or hopelessness, disturbed sleep or appetite, low energy, and poor concentration [21, 22]. Occurring at any age and affecting nearly twice as many women as men [23], depression is one of the most widespread of health conditions, affecting about 121 million people worldwide. Depression is expected to be second only to heart disease as the source of global burden of disease by 2020 [24, 25]. There is great potential for disrupting the lives of affected individuals, influencing their personal and family relationships, productivity in employment and personal lives, and ultimately, the community at large. As a social health condition, depression is linked to suicide, alcohol and drug misuse, and a variety of chronic, health impairing behaviors [26]. Likewise, depression can inhibit the effective management of illness and health promotion behaviors. 

Individuals living in rural areas are particularly vulnerable to depression. According to the 1999 National Health Interview Survey, 2.6 million rural adults in the USA suffer from depression [26]. The prevalence of major depression was found to be significantly higher among rural than among urban populations (6.11% compared with 5.16%) [27]. These statistics are particularly troubling given the underdetection and under-treatment of depression in rural primary care [28] and limited access to mental health professionals in the rural USA [29]. 

Stress results when external forces impact individuals and physical and behavioral responses are triggered. Any agent or stimulus that challenges adaptive capabilities can be considered a stressor [30], for example, a traumatic event or an illness. Postdisaster stress symptoms are often but not always reported more frequently by women than men [31]. It is important to recognize that uncontrollable and unpredictable stress takes a toll on physical and mental health, and that individuals' perception of the stressor and how they adapted to the stressful event are important factors that impact ability to cope with the stresses of illness [32]. Developing the capacity to manage stress is often helpful in dealing with the assaults of environmental stressors and the illnesses which they may trigger [33]. 

Realistic acceptance of illness is proposed to have a direct influence on how well individuals manage and adapt to living with their health condition [34]. Acceptance of illness is not resignation but integration of the disease into one's overall lifestyle; that is, “getting on with living.” Illness acceptance restores a sense of personal control by integrating the illness experience into the person's lifestyle. A strong relationship has been empirically demonstrated between illness acceptance and well-being, improved daily functioning and engagement in normal activities [35], higher quality of life, and better prognosis in a variety of chronic illnesses [36]. 

For persons exposed to a deadly toxin, such as those living in areas where amphibole asbestos-contaminated vermiculite was mined and processed, the probability of chronic illness is high along with its negative impact on psychosocial health. Thus, it is important to recognize the antecedents of negative psychosocial outcomes, such as depression, stress, and poor acceptance of illness, in this population and identify these individuals so that appropriate interventions and care management can be initiated. 

The research reported here was conducted to explore the biopsychosocial health status and health service needs of a national cohort of persons seeking care for exposure to Libby amphibole asbestos (LAA). The purposes of this paper are to (a) describe the psychosocial health status (depression, stress, and acceptance of illness) of the participants, and (b) explore differences in their psychosocial health status based on age, gender, residence, exposure pathway, insurance status, and access, availability, convenience, and financial aspects of health care among this sample. 

## 2. Material and Methods

A descriptive cross-sectional design was used for the study. Psychosocial health status and demographic data were collected via electronic and a paper-pencil questionnaire. The exposure pathway and physical health data were gleaned from the participants' health record. Approval for the study was granted by the Institutional Review Board at Montana State University. Data were collected from February through September, 2007, two years prior to the declaration of a public health emergency in Libby, Montana. Written informed consent was obtained, and coded, deidentified data were used in the analysis. Participants were clients of a specialty care clinic in Libby, Montana which has been providing asbestos health screening, follow-up health care, and consultation for exposed individuals since its opening in 2000. At the time of this study, the clinic was caring for more than 1500 patients from 32 states in the USA. 

To publicize the study, a notice was placed in the clinic newsletter and descriptive posters and brochures were displayed in the clinic waiting room. As clients presented to the clinic, they were asked to participate in the study. Those who consented completed either an electronic (via computer) or paper version of the questionnaire during their clinic visit. In addition, a group of individuals who were exposed to LAA and lived elsewhere in the USA, but continued to seek their specialty asbestos care at the clinic in Libby, were invited to participate in the study via normal clinic correspondence that included a packet containing a letter describing the study, consent form, and a paper copy of the questionnaire.

Data collected using the electronic methods were sent through a secure Internet connection to a protected database at the university research office. The paper/pencil data and health status information collected from the health record were electronically recorded and transmitted via the secure Internet site. Summary results from the psychosocial measures were reported back to the clinic weekly as they had a potential for impacting the plan of care. All questionnaire data collected became a part of the client's health record at the clinic.

### 2.1. Measures

The measures used to describe the psychosocial health status included Center for Epidemiological Studies-Depression Scale (CES-D) [37]; Perceived Stress Scale (PSS) [38]; Acceptance of Illness Scale (AOI) [34]; Patient Satisfaction Questionnaire (PSQ-III) [39]. Demographic questions were added to gather information on age, gender, education, marital status, and residence (local or distant). The exposure pathway (vermiculite worker/nonvermiculite worker), and primary health insurance coverage were gleaned from the health record. 

The CES-D is a 20-item self-report designed for initial screening in settings where psychological distress is not routinely assessed and is intended for use with research conducted in the general population [40]. Potential scores range from 0–60 with higher scores indicating higher levels of distress with a reported alpha ranging from  .84–.90 (current study 0.89). A score of 16 or greater suggests a clinically significant level of psychological distress, but not necessarily clinical depression and indicates that further psychological assessment may be necessary. 

The PSS consists of 14 items that measure the degree to which situations in one's life are seen as stressful [38] and is recommended by its developers as an outcome measure of levels of stress including that related to dealing with a chronic illness. Scores can range from 0 to 56 with higher scores indicative of higher perceived stress. The reported alphas ranged from  .84–.86, and in this study was 0.87. 

The AOIS was designed to measure persons' acceptance of chronic illness [34]. The 14-item scale includes statements related to acceptance of an illness such as “Having (this) disease is just part of life” and “I can't conquer (my disease) but I can adapt to it.” Potential scores range from 14 to 70 with higher scores indicating greater acceptance. The reported alpha was  .83 and slightly lower for our study at 0.76.

The PSQ-III is a survey, with seven subscales, designed to evaluate health services delivery [41]. The 12-item Access/Availability/Convenience subscale [39] was developed to measure these factors in relation to hospital, emergency, clinical, primary provider, and specialist care including convenience of services and nonfinancial access. Potential scores range from 12 (low) to 60 (high) and reliability is sufficient at  .86 (0.85 for this study). The financial aspects subscale of the PSQ-III contains eight items designed to address cost of care, health insurance, and ability to pay for care. Scores can range from 8 to 40 with higher scores indicative of greater satisfaction. The reported alpha was.80 (0.89 for this study).

### 2.2. Analytic Strategy

The purposes of this study were to (a) descriptively examine depression, stress, and acceptance of illness among persons seeking care for exposure to LAA, and (b) explore differences in those variables based on age, gender, education, asbestos exposure route, residence, health insurance status, and satisfaction with access and financial aspects of healthcare. Data were analyzed using SPSS (version 16). For the first purpose, means (observed) and standard deviations were produced to allow for general comparisons with other well and ill samples described in the literature. Minimal subgroup analysis based on age, gender, and education allowed for a more complete understanding of observed values. 

General linear modeling (GLM) was used to evaluate the second purpose with main effects as stated and a single a priori determined interaction of age∗gender. *P*-values for post hoc tests were adjusted using the Bonferroni correction with the exception of the age∗gender interaction where cell frequencies dropped, for example, *n* = 22 for women 0–49 years, *n* = 76 for women 50–64 years, leading to a violation of the equality of variance assumption and the use of the Games-Howell procedure. 

## 3. Results

A cohort of 386 patients of a specialty care clinic living in the Libby area, across Montana, and throughout the USA, with a history of environmental or occupational amphibole asbestos exposure, participated in the study. The majority were 50–64 years of age (46.9%); 17.9% had less than a high school education; 69.9% were married; and a slight majority were men (57.3%). Most resided in or near Libby, Montana (73.8%). The primary health insurance was from public sources such as the Centers for Medicare & Medicaid Services, Veterans Administration, and Social Security Administration (24.1%); private insurers (29%), and specialty asbestos health insurance called Health Network America (HNA)/Libby Asbestos Medical Plan (46.9%). 

The first study purpose was to describe the psychosocial health status defined by depression, stress, and acceptance of illness among patients (*N* = 386) exposed to LAA. On Table 1 are the means and standard deviations for measures of the psychosocial variables for the total sample, as well as subgroups created by age, gender, and education. For depression, stress, and acceptance of illness, the overall means were 13.5 (sd = 10.7), 21.0 (sd = 9.0), and 47.5 (sd = 7.5), respectively. More than one-third (34.5%) of the participants scored 16 or above on the depression scale, indicating a clinically significant level of psychological distress. 

**Figure 1:**
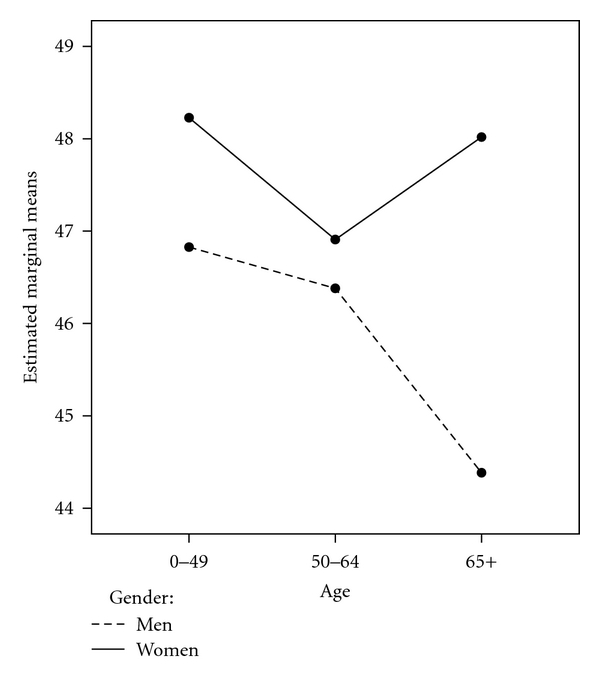
Acceptance of illness by age and gender.

The intent of the second study purpose was to explore differences in depression, stress, and acceptance of illness based on age, gender, education, asbestos exposure route, residence, insurance status, and satisfaction with access and financial aspects of healthcare. For depression, using an ANOVA analysis (Table 2), omnibus differences were found only for satisfaction with healthcare access *F*(3, 361) = 7.37, *P* < .05, and the age∗gender interaction *F*(2, 361) = 4.96, *P* < .05. There initially appeared to be an omnibus difference in depression based on primary insurance status, but in post hoc tests significant differences were not demonstrated for the three means. Multiple comparisons were performed for satisfaction with healthcare access and indicated that those scoring in the lowest quartile were significantly different than the 2nd, 3rd, and 4th quartiles on depression and that the 2nd and 3rd quartiles were different than the highest 4th quartile. Similarly, for the age∗gender interaction, group 6 (women aged 65+) was significantly different from all other groups of both women and men on depression. It is demonstrated in Figure 1 that for women in the oldest age category (65+), scores on depression dropped significantly from the next youngest age group of women and appear nearly 2 points lower than the lowest scores for men (50–64 years). 

**Figure 2:**
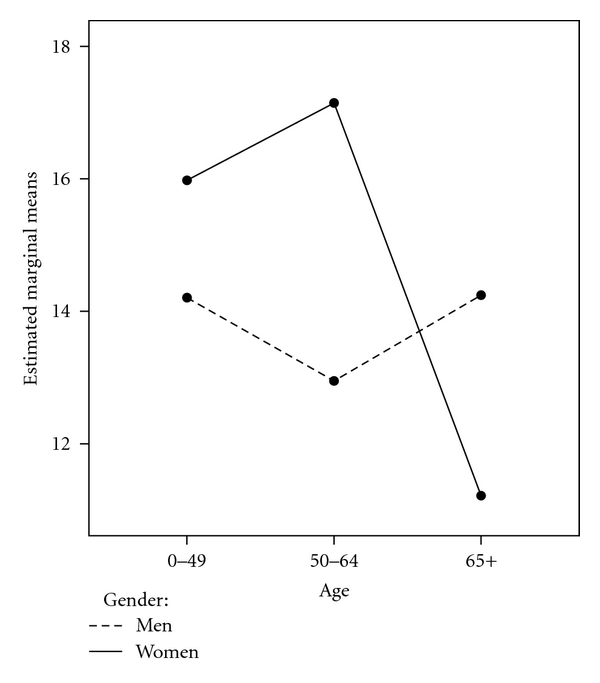
Depression by gender and age.

An identical GLM procedure was repeated examining stress with the same grouping variables and interaction effect of age∗gender (Table 3). Omnibus differences were found for satisfaction with financial resources *F*(3, 361) = 2.97, *P* < .05, satisfaction with healthcare access *F*(3, 361) = 5.48, *P* < .05, and the age∗gender interaction *F*(2, 361) = 4.81, *P* < .05. Multiple comparisons were performed for satisfaction with financial resources as well as healthcare access and indicated that those scoring in the lowest quartile were significantly different from the 3rd and 4th quartiles on stress. Additionally, for satisfaction with financial resources, the lowest quartile was different from the second quartile, and for both variables the 2nd and 4th quartiles differed. Mirroring the findings from depression, the age∗gender interaction showed that women in the 65+ age category differed from all other men and women on stress. These differences can be clearly seen in Figure 2 where the oldest women appear to score lowest on stress relative to the 5 other age groups. 

**Figure 3:**
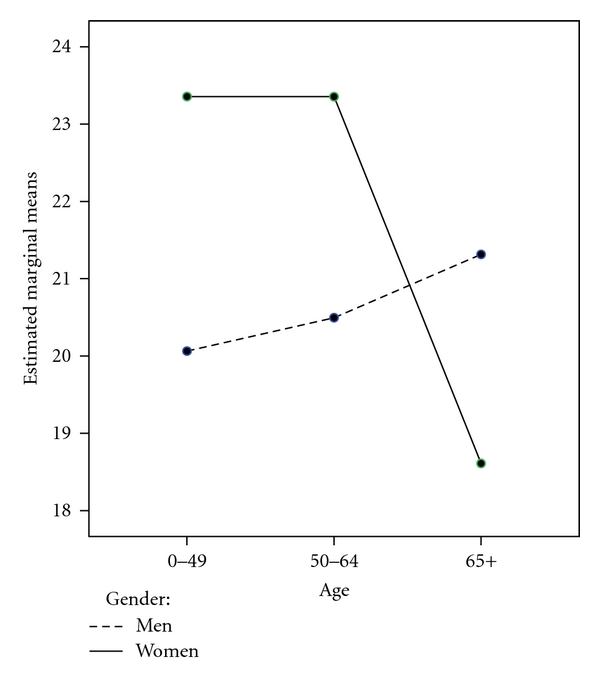
Stress by gender and age.

Last, the GLM procedure was repeated, examining acceptance of illness with the same grouping variables and interaction effect of age∗gender (Table 4). Omnibus differences were found for satisfaction with financial resources *F*(3, 361) = 7.93, *P* < .05 and the main effect of gender *F*(1, 361) = 4.20, *P* < .05. Multiple comparisons were performed for satisfaction with financial resources, and scores in the lowest and 2nd quartiles were significantly different from those in the 3rd and 4th quartiles on acceptance of illness. Although there was no significant age∗gender interaction, the main effect of overall differences between men and women can be seen in Figures 1, 2 and 3. At every age level, women had higher scores on acceptance of illness. 

## 4. Discussion

To our knowledge, this is the first exploration of the psychosocial health status of persons seeking treatment for exposure to LAA. For depression, stress, and acceptance of illness, no differences in means were found based on residence, exposure pathway, or insurance. Gender, age, and satisfaction with financial resources were significantly related to depression, stress, and acceptance of illness. Satisfaction with access to care was significant only for stress.

Based on the severity, length, and all-prevailing nature of the Libby environmental disaster, it could be anticipated that the overall depression level would be high. When comparing the mean depression scores of the Libby cohort to those for groups dealing with a serious chronic illness, such as HIV (*n* = 243; 17.4; sd = 13.5) [42], rheumatoid arthritis (*n* = 236; 16.6; sd = 13.1) [43], and chronic cough (*n* = 100; 18.3; sd = 13.2) [44], the Libby mean was at least three points lower. While the overall mean depression score for study participants was modest (13.5, sd = 10.7), more than one-third (34.5%) had scores indicating a clinically significant level of psychological distress. This is particularly striking when compared to the percent of Americans (5.4%) [45] and Montanans (6.7%) who reported being depressed [46]. Reported depression levels are often higher for women than for men; however, in our study the mean scores of women were not higher. 

The Libby cohort mean stress score was not exceptionally high. It had a lower overall stress score when compared to individuals with Parkinson's Disease (*n* = 70; 25.1; sd = 7.6) [47] and those with chronic obstructive pulmonary disease (COPD) (*n* = 181; 22.8; sd = 8.4) [48], and a slightly higher mean score than a group of women with an initial breast cancer diagnosis (*n* = 113; 18.1; sd = 6.9) [49]. Similar to the depression scores, the Libby study men and women had comparable mean scores for stress.

The impact of the disaster and the subsequent aftermath of resulting chronic illness and deaths in the community appear to be somewhat reflected in the scores on the AOI scale. Higher scores are indicative of a higher degree of acceptance. The Libby cohort had a lower mean acceptance score than a group of individuals with post-polio syndrome (*n* = 1603; 53.6; sd = 11.9) [50]. However, they were considerably higher than persons with diabetes (*n* = 59; 26.6; sd = 8.4) [51] and a group with multiple sclerosis (*n* = 786; 34.58; sd = 9.0) [34]. The Libby cohort may be struggling with accepting the effects of exposure to asbestos and ARD and integrating the residual limitations into their overall lifestyle because of the progressive nature of the illness. With progressive disease, new losses continue to emerge that must be accepted and integrated into a person's lifestyle. Individuals cannot plateau and establish a new equilibrium but must continually struggle with accepting the new state of their illness—similar to those dealing with conditions such as diabetes and multiple sclerosis. This score may also reflect the uncertainty about the long-term consequences, stigma, and loss of social support experienced by persons exposed to LAA [52, 53] and resulting chronic illness [54]. At every age, women in the Libby cohort had higher levels of acceptance than the men. This difference could be related to the working class rural culture of the community and men's traditional role and identity of providing for their families.

In the multivariable analysis, a cluster of variables was examined to explore potential differences in psychosocial indicator scores. The analysis for depression yielded two significant findings: those with higher satisfaction with health care access were less depressed; and age and gender had an interaction effect with older women being much less depressed than older men. For stress, those with higher satisfaction with health care access and with financial resources had lower levels of stress. Again, age and gender had an interaction effect with older women being less stressed than older men. For acceptance of illness, men were significantly different than women; however, differences based on age were not identified. As with stress, those with higher satisfaction with financial resources for health care had higher degrees of acceptance of their illness.

In evaluating the findings, it should be recognized that participants were recruited from a single specialty clinic and not from the Libby community at large. Recruiting from the community at large may have resulted in the inclusion of participants who were not associated with the clinic, were not being treated for LAA exposure or illness, and who may have had different health and illness experiences. Most participants were “local,” for example, recruited from Lincoln County and across Montana potentially limiting the ability to generalize the study findings to other groups of exposed persons.

## 5. Conclusion

In a study of patients seeking care for exposure to LAA, participants demonstrated moderate levels of stress and acceptance of illness however more than one-third (34.5%) had depression scores indicating a clinically significant level of psychological distress. Gender, age, and satisfaction with financial resources were significantly related to depression, stress, and acceptance of illness. Satisfaction with access to care was significant only for stress.

Depression can be reliably diagnosed and effectively treated by primary care providers [25]. The use of screening tools, similar to those used in this study, could be implemented in primary care practices serving populations living in or near environmental disaster areas around the world to assess psychosocial health. To take it one step further, incorporating a multidisciplinary team consisting of medical providers, nurses, and social workers could provide interventions to address individual, family, and community psychosocial issues. In our current study, this would include providing outreach, education, and referral services when needed for dealing with depression and chronic stress. Since depression can vary significantly across rural and urban areas and may reflect regional differences, demographic characteristics, socioeconomic conditions [46], and preventable social causes [55], additional research is necessary to understand the mental health issues facing additional cohorts of persons exposed to LAA. Of particular interest was the fact that, for a group of people living in an area with serious and persistent environmental contamination along with its associated health risks and potential sequelae, the reported stress levels were not particularly high. The study participants appeared to accept their illnesses with equanimity.

Further research is needed to gain a better understanding of the impacts of a disaster and its resulting health effects on men that specifically targets their personal identity and social roles. Understanding the resilience demonstrated by the women, a quality that might account for their adaptability, could be useful to health care providers and community-based nurses working with clients who live in an area affected by an environmental disaster. Studies are also needed that explore the psychosocial health status of exposed persons not affiliated with the specialty clinic in Libby to tease out the clinic providers' influence on the psychosocial health status and access and satisfaction with care.

Psychological distress identification, prevention, and intervention strategies, including self-management skills, are needed for persons exposed to environmental and workplace contamination such as the LAA disaster. With the declaration of a public health emergency in June of 2009, federal funding became available to address health issues resulting from the Libby environmental disaster; however, these resources were set up around a one-year recovery program as opposed to the long-term needs and duration of an ongoing environmental disaster. Population-based nurses and other health care providers are uniquely positioned to examine the biopsychosocial human response to an ongoing environmental disaster and intervene appropriately. They are obligated to work with the community to design and integrate best practice self-management interventions to reduce the effect of inordinate adversity from the event's lingering aftermath. 

## Figures and Tables

**Table 1:** Means for depression, stress, and acceptance of illness (*N* = 386).

	Depression	Stress	Acceptance of illness
	Mean (SD)	Mean (SD)	Mean (SD)
Overall	13.5 (10.7)	21.0 (9.0)	47.5 (7.5)

Age			
0–49	14.3 (14.1)	22.0 (9.2)	47.4 (7.2)
50–64	14.3 (11.0)	22.0 (9.4)	47.0 (7.4)
65+	12.2 ( 9.2)	19.3 (8.3)	48.3 (7.7)

Gender			
Men	13.2 (10.0)	21.0 (8.5)	46.7 (7.8)
Women	13.9 (11.7)	21.4 (9.6)	48.6 (8.0)

Education			
Less than high school	12.3 (9.3)	20.8 (8.7)	47.2 (8.6)
High school	13.8 (11.7)	21.2 (9.5)	48.0 (6.6)
More than high school	13.7 (10.3)	20.6 (8.7)	47.1 (7.8)

**Table 2:** Impact of external factors on depression scores (*N* = 386).

	Sum of squares	df	Mean square	*F*	*P*	Pair diffs.
Age						
0–49	293.24	2	146.62	1.46	.23	NA
50–64						
65+						

Gender						
Men	55.30	1	55.30	0.55	.46	NA
Women						

Education						
Less than high school	126.00	2	63.00	0.63	.53	NA
High school						
More than high school						

Exposure						
Worker	76.12	2	38.06	0.69	.69	NA
Family/HH contact						
Other						

Residence						
Local	35.89	1	35.89	0.36	.55	NA
Distant						

Primary insurance						
Public	796.84	2	398.42	3.97	.02	NA
Private						
HNA/LAMP						

Satisfaction with financial resources						
First quartile	421.63	3	140.54	1.40	.24	NA
Second quartile						
Third quartile						
Fourth quartile						

Satisfaction with healthcare access^1^						
First quartile (1)	2217.65	3	739.22	7.37	.00	1 versus 2 1 versus 3
Second quartile (2)						1 versus 4
Third quartile (3)						2 versus 4
Fourth quartile (4)						3 versus 4

Age∗Gender^2^						
Men 0–49 (1)	995.91	2	497.95	4.96	.00	1 versus 6
Women 0–49 (2)						3 versus 6
Men 50–64 (3)						5 versus 6
Women 50–64 (4)						2 versus 6
Men 65+ (5)						4 versus 6
Women 65+ (6)						

^1^Bonferroni adjusted *P* < .05.

^2^Games-Howell *P* < .05.

**Table 3:** Impact of external factors on stress scores (*N* = 386).

	Sum of squares	df	Mean square	*F*	*P*	Pair diffs.
Age						
0–49	205.06	2	102.53	1.46	.24	NA
50–64						
65+						

Gender						
Men	77.31	1	77.31	1.10	.30	NA
Women						

Education						
Less than high school	41.21	2	20.61	0.29	.75	NA
High school						
More than high school						

Exposure						
Worker	23.79	2	11.90	0.17	.85	NA
Family/HH contact						
Other						

Residence						
Local	233.64	1	233.64	3.32	.07	NA
Distant						

Primary insurance						
Public	164.20	2	82.10	1.17	.31	NA
Private						
HNA/LAMP						

Satisfaction-financial resources						
First quartile (1)	628.24	3	209.41	2.97	.03	1 versus 2
Second quartile (2)						1 versus 3
Third quartile (3)						1 versus 4
Fourth quartile (4)						2 versus 4

Satisfaction-healthcare access						
First quartile (1)	1159.14	3	386.38	5.48	.00	1 versus 3
Second quartile (2)						1 versus 4
Third quartile (3)						2 versus 4
Fourth quartile (4)						

Age∗Gender^2^						
Men 0–49 (1)	678.39	2	339.19	4.81	.01	1 versus 6
Women 0–49 (2)						3 versus 6
Men 50–64 (3)						5 versus 6
Women 50–64 (4)						2 versus 6
Men 65+ (5)						4 versus 6
Women 65+ (6)						

^1^Bonferroni adjusted *P* < .05.

^2^Games-Howell *P* < .05.

**Table 4:** Impact of external factors on acceptance of illness scores (*N* = 386).

	Sum of squares	df	Mean square	*F*	*P*	Pair diffs.
Age						
0–49	47.00	2	23.50	0.49	.61	NA
50–64						
65+						

Gender						
Men	202.17	1	202.17	4.20	.04	NA
Women						

Education						
Less than high school	13.86	2	6.93	0.14	.87	NA
High school						
More than high school						

Exposure						
Worker	121.33	2	60.66	1.26	.29	NA
Family/HH contact						
Other						

Residence						
Local	28.04	1	28.04	0.58	.45	NA
Distant						

Primary insurance						
Public	17.18	2	8.56	0.18	.84	NA
Private						
HNA/LAMP						

Satisfaction-financial resources						
First quartile (1)	1146.40	3	382.13	7.93	.00	1 versus 3
Second quartile (2)						1 versus 4
Third quartile (3)						2 versus 3
Fourth quartile (4)						2 versus 4

Satisfaction-healthcare access						
First quartile	290.98	3	96.69	2.01	.11	NA
Second quartile						
Third quartile						
Fourth quartile						

Age∗Gender						
Men 0–49	185.93	2	92.96	1.93	.15	NA
Women 0–49						
Men 50–64						
Women 50–64						
Men 65+						
Women 65+						

^1^Bonferroni adjusted *P* < .05.

## References

[1] Institute of Medicine (2003). *Preparing for the Psychological Consequences of Terrorism: A Public Health Strategy*.

[2] Najarian LM (2004). Disaster intervention: long-term psychosocial benefits in Armenia. *Prehospital and Disaster Medicine*.

[3] Culley MR, Zorland J, Freire K (2010). Community responses to naturally occurring asbestos: implications for public health practice. *Health Education Research*.

[4] Schneider A, McCumber D (2004). *An Air That Kills: How the Asbestos Poisoning of Libby, Montana Uncovered a National Scandal*.

[5] Levin SM, Kann PE, Lax MB (2000). Medical examination for asbestos-related disease. *American Journal of Industrial Medicine*.

[6] Horton K, Kapil V, Larson T, Muravov O, Melnikova N, Anderson B (2006). A review of the federal government’s health activities in response to asbestos-contaminated ore found in Libby, Montana. *Inhalation Toxicology*.

[7] Agency for Toxic Substances and Disease Registry (2002). *ATSDR Health Consultation for the Libby Community: Mortality in Libby, Montana, 1979–1998*.

[8] Peipins LA, Lewin M, Campolucci S (2003). Radiographic abnormalities and exposure to asbestos-contaminated vermiculite in the community of Libby, Montana, USA. *Environmental Health Perspectives*.

[9] Agency for Toxic Substances and Disease Registry (2002) *ATSDR Health Consultation For the Libby Community: Mortality in Libby, Montana, 1979 to 1998*.

[10] National Institute for Occupational Safety and Health (2008). *Work-Related Lung Disease (WoRLD) Surveillance System*.

[11] Agency for Toxic Substances and Disease Registry (2006). *Health Effects of Asbestos Exposure*.

[12] Whitehouse AC (2004). Asbestos-related pleural disease due to tremolite associated with progressive loss of lung function: serial observations in 123 miners, family members, and residents of Libby, Montana. *American Journal of Industrial Medicine*.

[13] U.S. Environmental Protection Agency (2007). *Libby Site Background*.

[14] Agency for Toxic Substances and Disease Registry (2002). *ATSDR Public Health Assessment*.

[15] U.S. Environmental Protection Agency (2000). *Asbestos Contamination in Vermiculite*.

[16] Lockey JE, Brooks SM, Jarabek AM (1984). Pulmonary changes after exposure to vermiculite contaminated with fibrous tremolite. *American Review of Respiratory Disease*.

[17] Rohs AM Pleural plaques in workers exposed to asbestiform contaminated vermiculite ore: a twenty-year follow-up.

[18] Baum A, Fleming I (1993). Implications of psychological research on stress and technological accidents. *American Psychologist*.

[19] Becker SM (1997). Psychosocial assistance after environmental accidents: a policy perspective. *Environmental Health Perspectives*.

[20] Palinkas LA, Lawrence A, Michael A (1993). Social, cultural, and psychological impacts of the Exxon Valdez oil spill. *Human Organization*.

[21] National Institute of Mental Health (2008). *Depression*.

[22] Winters CA, Cudney S, Sullivan T (2010). Expressions of depression in rural women with chronic illness. *Rural and Remote Health*.

[23] Mayo Clinic (2010). *Depression in Women: Understanding the Gender Gap*.

[24] Oreskovich J Serious psychological distress among Montana adults, 2007 BRFSS findings.

[25] World Health Organization (2011). Depression: what is depression?.

[26] Probst JC, Laditka SB, Moore CG (2006). Rural-urban differences in depression prevalence: implications for family medicine. *Family Medicine*.

[27] Probst JC, Laditka S, Moore CG (2005). *Depression in Rural Populations: Prevalence, Effects on Life Quality, and Treatment-Seeking Behavior*.

[28] Rost K, Fortney J, Zhang M, Smith J (1999). Treatment of depression in rural Arkansas: policy implications for improving care. *Journal of Rural Health*.

[29] Ng B, Bardwell WA, Camacho A (2002). Depression treatment in rural California: preliminary survey of nonpsychiatric physicians. *Journal of Rural Health*.

[30] Lawrence SA, Lawrence RM (1979). A model of adaptation to the stress of chronic illness. *Nursing Forum*.

[31] World Health Organization (2009). Gender, women and health: gender and disaster.

[32] Lazarus RS, Folkman S (1984). *Stress, Appraisal and Coping*.

[33] Cagle CS (2004). 3 themes described how self care management was learned and experienced by patients with chronic illness. *Evidence-Based Nursing*.

[34] Stuifbergen AK, Seraphine A, Roberts G (2000). An explanatory model of health promotion and quality of life in chronic disabling conditions. *Nursing Research*.

[35] Vowles KE, McCracken LM, Eccleston C (2008). Patient functioning and catastrophizing in chronic pain: the mediating effects of acceptance. *Health Psychology*.

[36] Richardson A, Adner N, Nordstrom G (2001). Persons with insulin-dependent diabetes mellitus: acceptance and coping ability. *Journal of Advanced Nursing*.

[37] Radloff LS (1977). The CES-D scale: a self-report depression scale for research in the general population. *Applied Psychological Measurement*.

[38] Cohen S, Kamarck T, Mermelstein R (1983). A global measure of perceived stress. *Journal of Health and Social Behavior*.

[39] Ware JE, Hays RD (1988). Methods for measuring patient satisfaction with specific medical encounters. *Medical Care*.

[40] Radloff LS (1977). The CES-D scale: a self-report depression scale for research in the general population. *Applied Psychological Measurement*.

[41] Ware JE (1983). Defining and measuring patient satisfaction with medical care. *Evaluation and Program Planning*.

[42] Bouhnik A-D (2005). Depression and clinical progression in HIV-infected drug users treated with highly active antiretroviral therapy. *Antiviral Therapy*.

[43] Escalante A, Del Rincon I, Mulrow CD (2000). Symptoms of depression and psychological distress among Hispanics with rheumatoid arthritis. *Arthritis Care and Research*.

[44] Dicpinigaitis PV, Tso R, Banauch G (2006). Prevalence of depressive symptoms among patients with chronic cough. *Chest*.

[45] Pratt LA, Brody DJ (2008). *Depression in the United States Household Population, 2005-2006*.

[46] Strine TW (2009). Metropolitan and micropolitan statistical area estimates of depression and anxiety using the patient health questionnaire-8 in the 2006 behavioral risk factor surveillance system. *International Journal of Public Health*.

[47] Frazier LD (2002). Stability and change in patterns of coping with Parkinson’s disease. *International Journal of Aging and Human Development*.

[48] Delgado C (2007). Sense of coherence, spirituality, stress and quality of life in chronic illness. *Journal of Nursing Scholarship*.

[49] Yang H-C, Brothers BM, Andersen BL (2008). Stress and quality of life in breast cancer recurrence: moderation or mediation of coping?. *Annals of Behavioral Medicine*.

[50] Stuifbergen AK, Harrison TC, Becker H, Carter P (2004). Adaptation of a wellness intervention for women with chronic disabling conditions. *Journal of Holistic Nursing*.

[51] Lewko J (2007). Quality of life and its relationship to the degree of illness acceptance in patients with diabetes and peripheral diabetic neuropathy. *Advances in Medical Sciences*.

[52] Cline R (2007). *In Your Own Words: A Preliminary Report of a Focus Group Study of Psychosocial Processes Associated with a Slow-Motion Environmental Disaster*.

[53] Cline RJW (2010). Community-level social support responses in a slow-motion technological disaster: the case of Libby, Montana. *American Journal of Community Psychology*.

[54] Mishel MH (1999). Uncertainty in chronic illness. *Annual Review of Nursing Research*.

[55] Lewis G, Booth M (1992). Regional differences in mental health in Great Britain. *Journal of Epidemiology and Community Health*.

